# Appearance Management in Korean Women Aged 60s Contributes to Ego Integrity and Quality of Life Beyond Youth

**DOI:** 10.1155/jare/5253003

**Published:** 2026-10-23

**Authors:** Ki Han Kwon, Heayoung Lim

**Affiliations:** ^1^ College of General Education Kookmin University Seoul South Korea kookmin.ac.kr; ^2^ College of General Education Namseoul University Cheonan South Korea nsu.ac.kr

**Keywords:** appearance management, cognitive age, ego integrity, older women, quality of life

## Abstract

**Aim:**

This study examined the relationship between appearance management motivation and quality of life in Korean women in their 60s and compared and analyzed the mediating effects of cognitive age identity and ego integrity.

**Methods:**

An online cross‐sectional survey was conducted with 350 women aged 60–69 living in the Seoul metropolitan area of Korea. Appearance management motivation, cognitive age identity, ego integrity, and quality of life were measured using validated scales. The indirect effect was mediated using 5000 bootstrap samples using Hayes’ PROCESS model.

**Results:**

Appearance management motivation had a direct positive effect on the quality of life (*β* = 0.297, *p* < 0.001), and two related pathways were identified. First, appearance management motivation significantly reduced cognitive age identity (*β* = −0.332, *p* < 0.001), and as cognitive age identity decreased, there was a tendency for the quality of life to increase (*β* = −0.261, *p* < 0.001). Second, appearance management motivation contributed to increasing ego integrity (*β* = 0.442, *p* < 0.001), and as ego integrity increased, the quality of life also significantly increased (*β* = 0.253, *p* < 0.001). The mediating effect of ego integrity (*β* = 0.112) was stronger than that of cognitive age identity (*β* = 0.087).

**Conclusion:**

In a society where appearance is important, appearance management suggests that it can be a psychological mechanism that goes beyond simply pursuing youthfulness and helps with self‐awareness, personal growth, and adaptation to the aging process. Appearance management that fosters self‐expression and personal capabilities can improve the quality of life of older people.

## 1. Introduction

The rapid aging of the population is having a profound impact on our society, and the socioeconomic role of the elderly is also being redefined. In line with this, voices are increasingly calling for changes in perceptions and response strategies for the elderly [1]. In 2020, the WHO declared the “UN Decade of Healthy Aging” with the aim of looking at aging in a new way and strongly urged active participation from all member states around the world [2]. Until now, policies and research on healthy aging have focused on physical health—including physical function, preventive medicine (lifestyle‐based practices for preventing chronic disease and functional decline), and dietary habits—but psychosocial aspects must also be given equal importance [3–5]. In fact, it has been shown that making lifestyle changes in advance, engaging in physical activity such as exercise, and other measures can significantly improve the quality of life across various population groups [6–9]. Of all the psychosocial factors, appearance management has settled as a particularly prominent behavioral pattern in a rapidly aging society like South Korea. Given that concerns about physical appearance are so widespread as people age [10], appearance management commonly becomes a self‐care practice used to maintain psychological well‐being and identity in old age [11–13].

The relationship between subjectively perceived age and quality of life has been revealed in various cultures and countries. Regardless of health or economic conditions, people who feel younger than their actual age have higher life satisfaction and experience mental well‐being [14]. Similar results have been observed in Korea [11, 15]. While demographic factors and chronic diseases and functional capacity have been the main subjects of research on factors that determine the quality of life of older adults [16, 17], there is a need to look at how proactive behaviors, such as appearance management, contribute to increasing life satisfaction [11, 18]. While clinical indicators are certainly important, taking proactive care of one’s own health is the base of psychological resilience and helps fill the gap between how old one feels and one’s actual physical aging [12, 19].

Korea is characterized by rapid aging and a sociocultural emphasis on youth. In this environment, middle‐aged women feel strong sociocultural pressure to remain young during the aging process and are actively adjusting their age identity to overcome this challenge wisely [10]. Women in their 60s grew up experiencing Korea’s rapid modernization firsthand. Against this backdrop, they have formed a new age perception that combines traditional concepts of old age with modern youth culture [10, 20]. In Korean society, appearance functions as an important element that shapes social relationships and status beyond the dimension of personal self‐expression [21]. Such “appearance‐focused culture” actually arises from collectivist values. A well‐groomed appearance is viewed as a sign of manners toward others and as evidence of taking good care of oneself [10, 22]. Korean women’s appearance management behaviors act as a core strategy for negotiating cognitive age identity. Late middle‐aged women seek to maintain their autonomy and sense of identity through appearance management, and this process has been shown to mitigate the psychological distress caused by physical aging [11, 23]. Lee and Kim revealed that “youth and health” are the most valuable aspects of life for Korean baby boomer women and reported that appearance management activities aimed at lowering cognitive age substantially increase life satisfaction [21].

According to Erikson’s psychosocial developmental stages, old age is the “ego integrity versus despair” stage, a period when individuals affirm life and realize its meaning [24]. Ego integrity is achieved when one integrates past life experiences and current physical changes into a single sense of self, rather than viewing them separately [25]. Meléndez et al. found that effective coping strategies have a positive impact on ego integrity [26]. Appearance management links the gap between older women’s inner selves and the physical changes brought on by aging, allowing them to rediscover their true selves in the process [11, 27]. This suggests that appearance management can serve as a coping mechanism that promotes psychological growth [28]. Although there has been active research on cognitive age and quality of life, little has been done to examine how appearance management affects ego integrity, a major psychosocial developmental stage in old age. Traditionally, appearance management among older women has been interpreted solely as a desire to maintain youth. However, the potential role of appearance management in identity formation and psychological development has been overlooked [29]. In this sense, appearance management can contribute to improving the quality of life by supporting ego integrity—the ability to integrate physical changes associated with aging with the internal developmental tasks of old age [11, 30].

Therefore, this study aims to (1) examine the relationship between appearance management motivation and quality of life among Korean women in their 60s and (2) analyze the comparative mediating effects of cognitive age identity and ego integrity in this relationship. Specifically, we seek to determine whether appearance management motivation affects ego integrity and how this pathway compares to the more established cognitive age identity pathway in terms of impact on quality of life. Through this approach, we aim to present a new paradigm for successful aging and improved quality of life in an aging society, while deepening understanding of psychosocial adaptation mechanisms among older women within Korea’s appearance‐conscious sociocultural context.

## 2. Methods

### 2.1. Participants and Procedure

This study conducted an online survey in December 2024, targeting 350 women aged 60–69 residing in the metropolitan area of Korea (Seoul, Gyeonggi, and Incheon). The participants aged 60–64 and those aged 65–69 were recruited in equal proportions to minimize bias. Participants were recruited through a professional online research company from its proprietary panel, and the sample size was determined using G∗Power analysis (effect size = 0.15, *α* = 0.05, and power = 0.90). We conducted the survey in strict compliance with the provisions of the Korean Statistics Act (Articles 8, 13, and 14). Accordingly, all participants were fully informed of the purpose of the survey and submitted written consent forms. All responses were gathered anonymously to ensure data quality, and the survey agency removed unreliable responses after conducting its own verification. Only the 350 respondents selected through this process were included in the final sample and used exclusively for this study.

### 2.2. Measures

In this study, “appearance management” refers not to invasive procedures such as plastic surgery but rather to daily grooming practices that encompass skincare, makeup, hair, nails, fashion, and weight management [11, 12]. The appearance management motivation scale was developed based on the “Behaviour Regulation in Exercise Questionnaire‐2 (BREQ‐2)” developed by Markland and Tobin to evaluate exercise motivation and the appearance management motivation derived from the studies by Leem [29, 31]. The scale demonstrated good validity (KMO = 0.871, Bartlett’s sphericity test *p* < 0.001) and reliability (Cronbach’s *α* = 0.886). Principal component analysis identified six factors, which explain approximately 70.4% of the total variance. Cognitive age identity was measured using the Cognitive Age Identity Scale developed by Barak and Schiffman. This scale measures Feel‐Age, Look‐Age, Do‐Age, and Interest‐Age, and in this study, age was divided into intervals and scored equitably, with 20s receiving 1 point and 90s receiving 8 points [32]. The scale showed good psychometric properties (KMO = 0.813, Bartlett’s sphericity test *p* < 0.001), with one factor explaining approximately 69.3% of total variance (Cronbach’s *α* = 0.851). We used the Northwestern Ego Integrity Scale to assess ego integrity. This scale evaluates the degree of psychological adaptation and developmental level in old age [33]. This scale demonstrated strong validity (KMO = 0.912, Bartlett’s sphericity test *p* < 0.001) and high internal consistency (Cronbach’s *α* = 0.906). One factor explained approximately 60.2% of the total variance. Quality of life was measured using the Korean version of WHOQOL‐BREF developed by Min [34]. The scale showed good psychometric properties (KMO = 0.905, Bartlett’s sphericity test *p* < 0.001), with four factors explaining approximately 71.5% of total variance (Cronbach’s *α* = 0.899). Except for cognitive age identity, all scales were measured using a 5‐point Likert scale. Statistics were calculated using SPSS WIN 25.0. Mean values and standard deviations were calculated using descriptive statistics, and correlations between variables were analyzed using correlation analysis. To verify the mediating effects of cognitive age identity and ego integrity between appearance management motivation and quality of life, Hayes’ (2020) PROCESS model 4 was implemented [35]. Indirect effects were estimated using 5,000 bootstrap samples and verified at significance levels of *p* < 0.05, *p* < 0.01, and *p* < 0.001.

## 3. Results

### 3.1. Participant Characteristics

Looking at the marital status, 78% were married, 12.9% were divorced or widowed, and 9.1% were single. By occupation, housewives were the most numerous at 50.3%, followed by office workers at 28.0%, others at 8.9%, self‐employed at 7.7%, and professionals at 5.1%. Education levels were distributed as college graduates or higher (55.5%), high school or lower (30.9%), and junior college (13.7%).

### 3.2. Descriptive Statistics and Correlations of Major Variables

As shown in the descriptive statistics analysis results in Table 1, all of the appearance management motivation (M = 3.41, SD = 0.53), cognitive age identity (M = 4.00, SD = 0.91), ego integrity (M = 3.70, SD = 0.67), and quality of life (M = 2.78, SD = 0.65) met the normal distribution conditions. Women in their 60s showed a high average value of appearance management motivation, confirming their activeness in appearance management. Cognitive age identity was low, suggesting that they consider themselves younger than their actual age.

**TABLE 1:** Descriptive statistics of major variables (*N* = 350).

Variable	M	SD	Min	Max	Skewness	Kurtosis
Appearance management motivation	3.41	0.53	1.25	4.50	−0.71	1.20
Cognitive age identity	4.00	0.91	1.50	7.25	0.43	0.91
Ego integrity	3.70	0.67	1.11	5.00	−0.91	1.87
Quality of life	2.78	0.65	1.00	4.89	0.31	0.21

*Note:* M = mean.

Abbreviation: SD, standard deviation.

In the correlation analysis in Table 2, appearance management motivation was inversely proportional to cognitive age identity and proportional to ego integrity and quality of life. There was a particularly significant negative correlation (*r* = −0.304, *p* < 0.01) between cognitive age identity and ego integrity. These results showed that women with lower cognitive age identity had higher ego integrity. The relationship between these two psychological factors provides important clues to understanding how appearance management affects quality of life.

**TABLE 2:** Correlations among major variables.

Variable	1	2	3	4
Appearance management motivation	1			
Cognitive age identity	−0.290^∗∗^	1		
Ego integrity	0.487^∗∗^	−0.304^∗∗^	1	
Quality of life	0.244^∗∗^	−0.272^∗∗^	0.337^∗∗^	1

^∗∗^ *p* < 0.01.

### 3.3. Mediating Effects of Cognitive Age Identity and Ego Integrity

Appearance management motivation had a direct positive effect on the quality of life (*β* = 0.297, *p* < 0.001). When analyzing the mediating role of cognitive age identity, appearance management motivation had a significant negative effect on cognitive age identity (*β* = −0.332, *p* < 0.001), and cognitive age identity had a negative effect on the quality of life (*β* = −0.261, *p* < 0.001). When controlling cognitive age identity, the direct effect of appearance management motivation on the quality of life remained significant but slightly decreased (*β* = 0.211, *p* < 0.001). Similarly, when analyzing the mediating role of ego integrity, appearance management motivation had a significant positive effect on ego integrity (*β* = 0.442, *p* < 0.001), and ego integrity had a positive effect on the quality of life (*β* = 0.253, *p* < 0.001). When controlling ego integrity, the direct effect of appearance management motivation on the quality of life was significant but reduced (*β* = 0.185, *p* < 0.001). Table 3 reveals the results of the mediation effect analysis.

**TABLE 3:** Mediating effects of cognitive age identity and ego integrity in the relationship between appearance management motivation and quality of life in Korean women in their 60s.

Variable			*β*	*t*	*R* ^2^	*F*	LLCI	ULCI
Independent		Dependent						
AM Motivation	⟶	Quality of life	0.297^∗∗∗^	8.232	0.088	67.766^∗∗∗^	0.271	0.441
AM Motivation	⟶	CAI	−0.332^∗∗∗^	−9.313	0.111	86.740^∗∗∗^	−0.667	−0.435
AM Motivation	⟶	Quality of life	0.211^∗∗∗^	5.684	0.149	61.150^∗∗∗^	0.165	0.339
CAI			−0.261^∗∗∗^	−7.057			−0.241	−0.136
AM Motivation	⟶	Quality of life	0.297^∗∗∗^	8.232	0.088	67.766^∗∗∗^	0.271	0.441
AM Motivation	⟶	Ego integrity	0.442^∗∗∗^	13.027	0.196	169.711^∗∗∗^	0.469	0.636
AM Motivation	⟶	Quality of life	0.185^∗∗∗^	4.735	0.140	56.804^∗∗∗^	0.130	0.314
Ego Integrity			0.253^∗∗∗^	6.471			0.169	0.316

*Note: β*: standardized regression coefficient, *t*: *t*‐value, *R* ^2^: coefficient of determination, *F*: *F*‐statistic. LLCI: lower limit of 95% confidence interval, ULCI: upper limit of 95% confidence interval.

Abbreviations: AM Motivation, Appearance Management Motivation; CAI, cognitive age identity.

^∗∗∗^ *p* < 0.001.

When the statistical significance of the two indirect effects was confirmed by bootstrap analysis, it was found that cognitive age identity and ego integrity partially mediated the relationship between appearance management and quality of life. Notably, the standardized indirect effect mediated by ego integrity (*β* = 0.112) was stronger than the effect mediated by cognitive age identity (*β* = 0.087), suggesting that appearance management motivation exerts a stronger influence on the quality of life by enhancing ego integrity through cognitive age identity. According to Cohen’s guidelines, the ego integrity pathway (*β* = 0.112) meets the medium effect size threshold, while the effect of cognitive age identity (*β* = 0.087) is close to it, reflecting the substantial importance of appearance management’s influence [36]. Details of the indirect effects are presented in Table 4.

**TABLE 4:** Verification of indirect effects of cognitive age identity and ego integrity in the relationship between appearance management motivation and quality of life.

Variable	Path	*B*	se	*β*	LLCI	ULCI
Cognitive age identity	Total effect (*β*)	0.356	0.043	0.297	0.271	0.441
	Direct effect (*β*)	0.252	0.044	0.211	0.165	0.339
	Indirect effect (*β*)	0.104	0.023	0.087	0.062	0.150

Ego integrity	Total effect (*β*)	0.356	0.043	0.297	0.271	0.441
	Direct effect (*β*)	0.222	0.047	0.185	0.130	0.314
	Indirect effect (*β*)	0.134	0.026	0.112	0.086	0.185

*Note: B*: unstandardized regression coefficient, *β*: standardized regression coefficient, LLCI: lower limit of 95% confidence interval, ULCI: upper limit of 95% confidence interval.

Abbreviation: SE, standard error.

Figure 1 is a path diagram illustrating the relationships among appearance management motivation, cognitive age identity, ego integrity, and quality of life. It also shows the relative influence of cognitive age identity and ego integrity.

**FIGURE 1:**
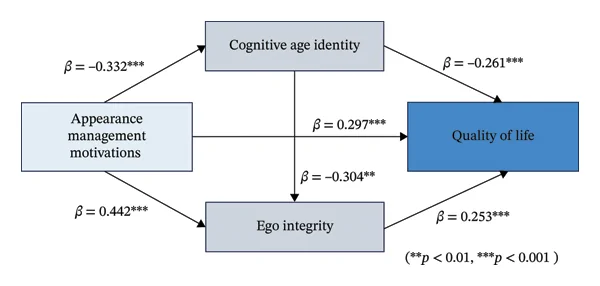
The impact of appearance management motivation on the quality of life in Korean women in their 60s: mediating effects of cognitive age identity and ego integrity.

## 4. Discussion

The Korean sociocultural context can greatly help us understand the empirical findings on the two pathways through which appearance management motivation is related to the quality of life. Although this association was only moderate, it was statistically significant. This implies that the quality of life is a concept in which physical, mental, and social factors are intricately intertwined [37, 38]. Therefore, appearance management may be viewed as a significant factor among the various elements that contribute to healthy aging [37]. Upon considering these various factors, results from this study indicate that appearance management motivation has a greater impact on the quality of life through ego integrity (*β* = 0.442) than through cognitive age identity (*β* = −0.332).

The results of this study challenge the conventional wisdom that appearance management among women in their 60s begins with a simple desire to look younger. This finding is particularly significant given the unique context of Korean society, where rapid population aging and youth‐oriented values persist, and where late‐middle‐aged women are experiencing the challenge of identity exploration [10, 21]. As shown in the study by Ehlinger‐Martin et al. [39], the gap between subjective age identity and appearance begins to appear in the mid‐30s. This study adds to the significance by showing how Korean women in their 60s transform this subtle tension into a positive force [40].

The stronger mediating effect of ego integrity (*β* = 0.112) compared to cognitive age identity (*β* = 0.087) suggests that appearance management functions as a form of adaptive coping mechanism. According to a study by Lee and Lee, 70% of Korean women use appearance management to cope with changes due to menopause [20]. Our study shows the psychological pathways through which this phenomenon occurs. The finding of a study by Rahman and Chang that baby boomer women actively manage their appearance while accepting physical changes of aging is consistent with the correlation between cognitive age identity and ego integrity (*r* = −0.304) in our data [40]. This relationship suggests that Korean women in their 60s understand appearance management as part of a broader developmental process of pursuing self‐acceptance and life satisfaction rather than simply trying to look younger. In the appearance‐oriented culture of Korean society, it can be seen as an adaptive strategy that reflects the cultural specificity of aging [10, 20].

Previous studies have primarily focused on the perspective that “looking young makes one feel good” [28], overlooking the fundamental psychological mechanisms underlying appearance management [11]. This study verified the mediating effect of cognitive age identity in the relationship between appearance management and well‐being identified in previous studies, but it is significant that the mediating effect of ego integrity was stronger [28]. This study provides empirical evidence that the motivation for appearance management in women in their 60s has a positive effect on the quality of life through ego integrity and provides a new perspective on the existing view that older women manage their appearance by succumbing to social esthetic standards or pressure. This study broadened our understanding of the aging process of older women by revealing what psychological mediators work beyond the relationship between appearance management motivation and quality of life [37, 41, 42]. This suggests that older Korean women can express themselves and enhance their abilities through appearance management to improve their quality of life. This study focused on appearance management as an intervention to improve the quality of life in old age, responding to the need for integration of individual and sociocultural factors [43].

This study provides practical implications for Korean society. Community‐supported appearance management education programs and beauty services can help improve the quality of life by increasing positive self‐perception, self‐esteem, and ego integrity in older women [44]. Approaches to older women should focus on authentic self‐expression and finding deeper meaning in life. The results of this study support the multidimensional framework for healthy aging by Kamide et al., suggesting that physical, mental, and social factors work together harmoniously to improve the well‐being of older people [37, 38]. Appearance management may help support healthy aging just as much as a regular exercise regime. Recently, studies on Pilates [6] and walking [9] have shown that these activities improve both physical and mental health as well as overall well‐being. Clinically, these findings indicate that, if appearance management is included as one pillar of lifestyle medicine (an evidence‐based field that modifies lifestyle behaviors to prevent, manage, and reverse chronic conditions) [8], it may serve as a form of support that helps both individuals’ inner life and their social participation [45].

## 5. Conclusion

This study verified that the appearance management motivation of Korean women in their 60s has a direct and positive effect on the quality of life and contributes indirectly through cognitive age identity and ego integrity. The mediating effect of ego integrity (*β* = 0.112) was stronger than that of cognitive age identity (*β* = 0.087), indicating that appearance management among older women goes beyond the conventional view of simply maintaining youth or succumbing to sociocultural pressures. Instead, it serves as a psychological mechanism contributing to ego integrity, which is associated with the meaning and wisdom of life during old age. In South Korea, a society with a strong appearance‐oriented culture, these findings are particularly significant. Amid a societal emphasis on appearance as a means of social acceptance, older women are transforming appearance management from a form of social conformity into a psychological resource to enhance their sense of life meaning. This study has the strength of revealing how Korean women in their 60s gain psychosocial strengths, such as autonomy and resilience, through appearance management [11, 20, 23]. By viewing appearance management not merely as a matter of beauty but as a process of developmental growth, this study lays the theoretical and practical foundation for a self‐expression‐centered appearance management program designed to support the psychological well‐being of Korean women in their 60s.

This study has several limitations. Results are difficult to generalize because the study was conducted only among women living in Seoul and the metropolitan area. Living in a particular region has a significant impact on one’s lifestyle, but a sample concentrated in the Seoul metropolitan area lacks regional diversity [46]. In addition, the results of this study were obtained through a single online quantitative survey, which limits our understanding of deep causal relationships. Future studies should adopt longitudinal designs and include participants from a wider range of regions to improve the generalizability of the findings. In addition, combining qualitative approaches can yield more varied and in‐depth insights into different appearance management practices. Such research will contribute to deepening our understanding of the psychological effects of appearance management on the quality of life of older women. In addition, we plan to conduct cultural comparative studies between Korea and other countries to distinguish between universal aspects and cultural specificity in the value of appearance management.

## Funding

This research did not receive any specific funding from public, commercial, or non‐profit organizations.

## Ethics Statement

This study was conducted following ethical guidelines. All participants provided written informed consent prior to participation. Research procedures adhered to privacy regulations under the Statistical Act (Articles 8, 13, and 14) of the Republic of Korea. Ethical review and approval were waived for this study by Institution Committee due to Legal Regulations Pursuant to Article 15 (2) of the Bioethics and Safety Act and Article 13 of its Enforcement Rules (promulgated by the Ministry of Health and Welfare, Republic of Korea, and administered by the Korea Ministry of Government Legislation).

## Conflicts of Interest

The authors declare no conflicts of interest.

## Supporting Information

Additional supporting information can be found online in the Supporting Information section.

## Supporting information


**Supporting Information** STROBE Statement—Checklist of items that should be included in reports of cross‐sectional studies.

## Data Availability

The data supporting this study’s findings are available from the corresponding author upon reasonable request.
