# Supplementary material for: Appearance Management in Korean Women Aged 60s Contributes to Ego Integrity and Quality of Life Beyond Youth
Source: J Aging Res. 2026 Oct 23;2026:5253003. doi: 10.1155/jare/5253003 (PMC13692834; doi:10.1155/jare/5253003)
Supplement: Supplementary file 1 [file JARE-2026-5253003-s001.doc]

STROBE Statement—Checklist of items that should be included in reports of ***cross-sectional studies***

|  | Item No | Recommendation |
| --- | --- | --- |
| **Title and abstract** | 1 | (*a*) **Page 1,** Abstract clarify the "online cross-sectional survey" design. |
|  |  | (*b*) **Page 1 (Abstract):** Objective, methods, main results, and conclusion are balanced. |
| Introduction | | |
| Background/rationale | 2 | **Page 2 (Para 1-4):** Explains the sociocultural pressure on appearance in Korea, the transition to old age, and the role of psychological resilience. |
| Objectives | 3 | **Page 2 (Para 5):** States the aim to examine the relationship between Appearance management motivation and quality of life, mediating effects of cognitive age identity and, ego integrity. |
| Methods | | |
| Study design | 4 | **Page 3 (Participants and Procedure):** Mentions "online cross-sectional survey." |
| Setting | 5 | **Page 3 (Participants and Procedure):** Online survey conducted in December 2024 in the Seoul metropolitan area. |
| Participants | 6 | (*a*) **Page 3 (Participants and Procedure):** Women aged 60–69. Quota sampling (50:50 ratio) for early and late 60s. |
| Variables | 7 | **Page 3-4 (Measures):** Defines Appearance management motivation (independent), cognitive age identity and ego integrity (mediators), and quality of life (dependent). |
| Data sources/ measurement | 8* | **Page 3-4 (Measures):** Uses validated scales: BREQ-2 (Appearance management motivation), Cognitive Age Identity Scale, NEIS (ego integrity), and WHOQOL-BREF (quality of life). |
| Bias | 9 | **Page 3 (Participants and Procedure):** Recruitment through proprietary panels, quota sampling, and anonymous responses |
| Study size | 10 | **Page 3 (Participants and Procedure):** Determined by G*Power analysis (effect size 0.15, power 0.90, N=350). |
| Quantitative variables | 11 | **Page 3 (Measures):**  All variables were measured on 5-point Likert scales. Cognitive age was categorized into age intervals and scored from 20s (1 point) to 90s (8 points). |
| Statistical methods | 12 | (*a*) **Page 4 (Measures):** Describes use of SPSS WIN 25.0, descriptive statistics, correlation analysis, and Hayes' (2020) PROCESS Model 4 with 5,000 bootstrap samples to test mediating effects. |
|  |  | (*b*) **Page 3-4 (Mediating Effects section):** Compares the relative influence of two mediators (cognitive age identity and ego integrity); no other subgroup analyses conducted. |
|  |  | (*c*) **Page 3 (Participants and Procedure):** Unreliable responses removed; 350 complete cases retained. |
|  |  | (*d*) **Not applicable** |
|  |  | (*e*) **Not applicable** |
| Results | | |
| Participants | 13* | (a) **Page 4 (Para 1):** Confirms 350 final participants after internal quality screening. |
| Descriptive data | 14* | **Page 4 and Table 1:** Reports demographic features (marital status, occupation, education level) and descriptive stats (M, SD, Skewness, Kurtosis). |
| Outcome data | 15* | **Page 4-5 (Mediating Effects section) and Tables 3–4:** Reports the direct, indirect, and total effects of the mediation analysis. |
| Main results | 16 | **Page 4–5 (Mediating Effects section) and Tables 3–4:** Reports effect estimates (β), statistical significance, and 95% confidence intervals. |
| Other analyses | 17 | Not applicable |
| Discussion | | |
| Key results | 18 | **Page 5 (Para 1):** Summarizes the findings regarding the dominant role of ego integrity over cognitive age. |
| Limitations | 19 | **Page 6~7 (Conclusion, Para 2):** Discusses regional concentration in the Seoul metropolitan area and the cross-sectional single-survey design as limitations. |
| Interpretation | 20 | **Page 5~6 (Para 2~4):** Interprets findings through the lens of identity reconstruction and multidimensional aging (Kamide et al., 2025). |
| Generalisability | 21 | **Page 6 (Conclusion, Para 2):** Addresses the difficulty of generalising results to non-metropolitan or rural populations. |
| Other information | | |
| Funding | 22 | **Page 7 (Funding statement):** Declares no specific funding was received. |

*Give information separately for exposed and unexposed groups.

**Note:** An Explanation and Elaboration article discusses each checklist item and gives methodological background and published examples of transparent reporting. The STROBE checklist is best used in conjunction with this article (freely available on the Web sites of PLoS Medicine at http://www.plosmedicine.org/, Annals of Internal Medicine at http://www.annals.org/, and Epidemiology at http://www.epidem.com/). Information on the STROBE Initiative is available at www.strobe-statement.org.
